# Synthesis, Giant Dielectric, and Pyroelectric Response of [001]-Oriented Pr^3+^ Doped Pb(Mg_1/3_Nb_2/3_)O_3_-PbTiO_3_ Ferroelectric Nano-Films Grown on Si Substrates

**DOI:** 10.3390/ma11122392

**Published:** 2018-11-28

**Authors:** Changlong Cai, Deqiang Zhang, Weiguo Liu, Jun Wang, Shun Zhou, Yongming Su, Xueping Sun, Dabin Lin

**Affiliations:** 1 Thin Film and Optical Manufacturing Technology, Key Laboratory of Ministry of Education, Xi’an Technological University, Xi’an 710032, China; cmcwlh@163.com (C.C.); zhangdqiang@163.com (D.Z.); wgliu@163.com (W.L.); zsemail@126.com (S.Z.); yongmingsu@126.com (Y.S.); sunxueping.1988@163.com (X.S.); 2 Department of Basic Science, Air Force Engineering University, Xi’an 710051, China; wangjun563@163.com

**Keywords:** thin film, pyroelectric, ferroelectric, dielectric, sol–gel

## Abstract

The [001]-oriented Pr^3+^ doped Pb(Mg_1/3_Nb_2/3_)O_3_-0.30PbTiO_3_ (Pr-PMN-PT) thin films with a composition near the morphotropic phase boundary (MPB) were synthesized by a sol–gel method. The crystal structure was characterized using X-ray diffraction. It was found that a single perovskite phase was achieved in Pr-PMN-PT thin films annealed at 650 °C for 3 min. The dielectric constant (*ε*_r_) value was 2400 in 2.5% Pr-PMN-PT thin films at room temperature, 110% higher than that of pure PMN-PT samples. Through 2.5% Pr^3+^ doping, remanent polarization (*P*_r_) and coercive field (*E*_c_) values increased from 11.5 μC/cm^2^ and 35 kV/cm to 17.3 μC/cm^2^ and 63.5 kV/cm, respectively, in PMN-PT thin films. The leakage current densities of pure and 2.5% Pr-PMN-PT thin films were on the order of 1.24 × 10^−4^ A/cm^2^ and 5.8 × 10^−5^ A/cm^2^, respectively, at 100 kV/cm. A high pyroelectric coefficient (*p*_y_) with a value of 167 μC/m^2^K was obtained in 2.5% Pr-PMN-PT thin films on Si substrate, which makes this material suitable for application in infrared detectors.

## 1. Introduction

Pb(Mg_1/3_Nb_2/3_)O_3_-PbTiO_3_ (PMN-PT) bulk piezoelectric materials with compositions near the morphotropic phase boundary have attracted much attention as a candidate piezoelectric material for device application in the fields of microelectromechanical system (MEMS) and nanoelectromechanical systems (NEMS) [1,2,3]—such as infrared (IR) detectors [4], cantilever-type piezoelectric devices [5,6], multilayer capacitors [7], and ultrasonic transducers [8,9,10]—due to the high field strain (1.7%) [11], pyroelectric coefficient (~1500 μC/m^2^K) [12], and longitudinal coupling factor (~95%) [13,14]. Especially for uncooled IR detectors, pyroelectricity is indeed one of the most promising principles. Compared with bulk crystals, thin films have the advantage of low-cost, reduced material consumption, small cell size, energy efficiency, and ease of integration in Si semiconductor technology. For PMN-based bulk crystals, the pure perovskite structure can easily be synthesized using the two-step columbite precursor method [15,16,17]. On the contrary, the second pyrochlore phase in PMN-PT thin films is related to many factors, for example annealing temperature/time [18], sample thickness [19], excess Pb content [20,21], etc., which restrict the pyroelectric, piezoelectric, and dielectric properties in PMN-PT thin films.

Many methods and approaches—such as radio frequency (RF) magnetron sputtering [22], pulsed laser deposition (PLD) [4], chemical vapor deposition (CVD) [23], chemical solution deposition (CSD) [24], and so forth—have been adopted to fabricate high-quality PMN-PT thin films. Sol–gel processing, a kind of chemical solution deposition, has many advantages that make it suitable for obtaining high-quality PMN-PT thin films on Si substrate for industrial applications [25,26]. Dielectric constants and remanent polarization in slim P-E hysteresis loops were on the order of 1692 and 13.31 μC/cm^2^ respectively in the PMN-0.30PT thin films using sol-gel technology [27]. Recently, a giant pyroelectric response (−550 μCm^−2^K^−1^) driven by the electric field was obtained in 0.68Pb(Mg_1/3_Nb_2/3_)O_3_-0.32PbTiO_3_/Ba_0.5_Sr_0.5_RuO_3_ hetero structures grown on [110]-oriented NdScO_3_ single crystals by pulsed-laser deposition (PLD) [4] indicating good potential for low dimensional pyroelectric devices.

Rare earth elements such as Er^3+^, La^3+^, and so forth have been used to improve the up-conversion photoluminescence in bulk piezoelectric materials [28,29]. Recently, an ultrahigh piezoelectric coefficient (*d*_33_ = 1500 pC/N) was obtained in Sm-doped PMN-PT polycrystalline [30] based on polar nano-region (PNR) design in relaxor based ferroelectric materials. For Pb(Zr,Ti)O_3_ (PZT) thin films, the dielectric and piezoelectric properties were improved by La doping [31]. Until now, little research has focused on the pyroelectric or piezoelectric properties of rare earth element-doped relaxor-PT (PMN-PT or PIN-PMN-PT) thin films. With the aim of low cost and high performance materials for MEMS device fabrication, the Pr^3+^ doped PMN-PT thin films were prepared by the sol–gel method at various annealed temperatures and times. The phase structure of the samples was analyzed by X-ray diffraction. Then, the surface morphology of Pr-PMN-PT thin films was measured by scanning electron microscopy, atomic force microscopy, and piezoresponse force microscopy. Finally, dielectric, pyroelectric, and ferroelectric properties of Pr-PMN-PT thin films were investigated.

## 2. Materials and Methods

Before sol–gel processing, a TiO_2_ diffusion barrier layer was first fabricated by oxidizing the Ti metal layer as follows: Ti films of 60 nm thickness were deposited on SiO_2_/Si (100) substrate by DC magnetron sputtering in an Ar gas environment at room temperature. The sputtering power was 175 W. The base vacuum of Ti sputtering was higher than 8 × 10^−4^ Pa. The ratio of Ar:O_2_ was 90:10. After deposition, the Ti layer was annealed in a rapid thermal processing (RTP) furnace at 700 °C for 1 h. Then, a Pt bottom electrode of 80 nm thickness was deposited on the TiO_2_ diffusion barrier layer. The sputtering parameters were the same as the Ti metal layer deposition. The sheet resistance was about 2.85 Ω/sq, which was measured by the four probe method.

After that, Pr^3+^ doped Pb(M_1/3_Nb_2/3_)O_3_-0.30PbTiO_3_ (xPr-(1 − x)(PMN-0.30PT), x = 1%, 2%, 2.5%, and 3%) precursor solutions were prepared by the sol–gel method. The lead acetate trihydrate (Pb(CH_3_COO)_2_·3H_2_O), magnesium acetate tetrahydrate (Mg(CH_3_COO)_2_·4H_2_O), niobium ethoxide (Nb(OCH_3_CH_2_)_5_), tetrabutyl titanate (Ti(OC_4_H_9_)_4_), and praseodymium acetyl acetone (C_15_H_21_PrO_6_·xH_2_O) were used as starting materials to synthesize the Pr-PMN-PT sol–gel solution. An additional 20 mol% of lead acetate trihydrate was added to equalize the Pb volatilization and 5 mol% more magnesium acetate tetrahydrate was mixed to boost the production of perovskite phase. Next, 2-methoxyethanol (C_5_H_8_O_2_) and glacial acetic acid (CH_3_COOH) were added as solvent and catalyst respectively. Acetyl acetone (C_5_H_8_O_2_) was added as a stabilizer in the Pb-Pr-Mg-Nb-Ti solution. The concentration of the Pr-PMN-PT solution was attenuated to 0.4 mol/L. Then, the Pr-PMN-PT thin films were deposited by spin coating on Pt/TiO_2_/SiO_2_/Si substrates at 2000 rpm for 9 s before the spin coating was subsequently sped up to 4000 rpm for 20 s. To evaporate the solvent in solution, the sample was dried at 220 °C for 3 min after each deposition and then pyrolyzed at 450 °C for 5 min. Finally, Pr-PMN-PT films were annealed at 600, 650, and 700 °C for 1–5 min in air by RTP at ramp rates +20 °C/s and −5 °C/s respectively. In order to get 500 nm thick film, this entire process was repeated 10 times. For further electric measurement, Au top electrodes were deposited on Pr-PMN-PT samples using magnetron sputtering through a shadow mask with a 1 mm diameter.

The crystalline structure of Pr-PMN-PT thin films was analyzed by X-ray diffraction (XRD) (Shimadzu X-6000, Kyoto, Japan) with Cu-Kα radiation in the 2theta range of 20°–60° and a scan step length of 0.02 at room temperature. The surface morphologies of the samples were characterized by a field emission scanning electron microscope (FE-SEM) (ZEISS Gemini SEM 500, Jena, Germany), atomic force microscope (AFM) (Bruker Multimode 8, Billerica, MA, USA), and piezoresponse force microscopy (PFM) (Bruker dimension icon, Billerica, MA, USA). For electric measurement, the samples were all of a pure perovskite phase structure due to the high electric performance. The dielectric properties were measured using an impedance analyzer (HP 4284, Hewlett Packard, Palo Alto, CA, USA) with a precision LCR meter connected to a heating stage (Linkam THMSE 600, Waterfield, UK). The P-E hysteresis loops and leakage current were examined by a standard ferroelectric system (aixACCT TF-2000, Aachen, Germany). The pyroelectric coefficient (*p*_y_) was calculated by the equation as below:

(1) $$p_{y}\left(T\right)=\frac{I}{A\frac{dT}{dt}}\;\left(\mu C/m^{2}K\right),$$

where *I* is the pyroelectric current, *A* is the surface area of the samples, and *dT*/*dt* is the rate of temperature cooling.

## 3. Results and Discussion

### 3.1. Phase Structure

**Figure 1:**
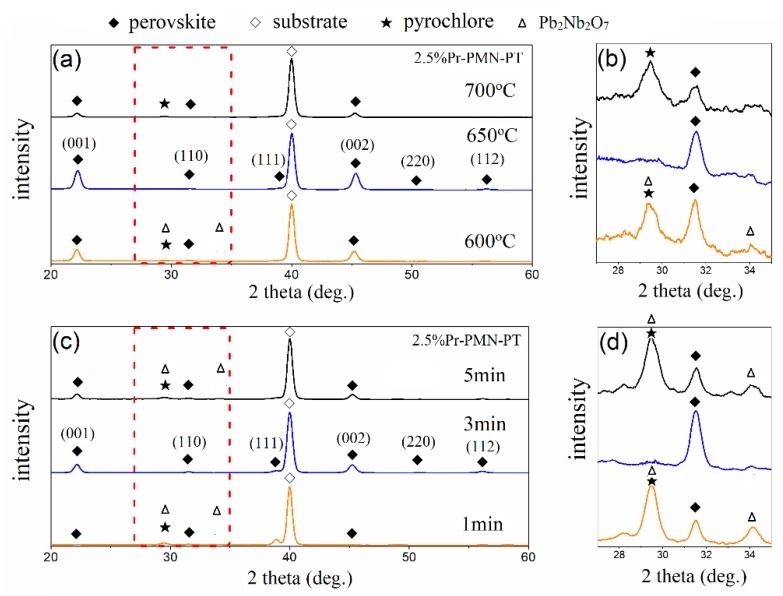
XRD patterns of 2.5% Pr^3+^ doped Pb(Mg_1/3_Nb_2/3_)O_3_-0.30PbTiO_3_ (Pr-PMN-PT) thin films as a function of annealing temperatures between 600 °C and 700 °C: (**a**,**b**); XRD patterns of 2.5% Pr-PMN-PT thin films annealed at 650 °C as a function of annealing times between 1 min and 5 min: (**c**,**d**).

Avoiding the formation of a pyrochlore phase is a key factor for the synthesis of PMN-PT thin films as the addition of this phase will reduce the piezoelectric and ferroelectric properties of the resulting thin films. In order to restrict the formation of a pyrochlore phase, the annealing conditions for thin film preparation were first investigated. Figure 1a,b show the X-ray diffraction patterns of 2.5% Pr^3+^ doped PMN-0.30PT thin films deposited on Pt/TiO_2_/SiO_2_/Si substrates annealed at 600 °C, 650 °C, and 700 °C for 2 min. The perovskite phase peaks were observed at 2θ = 22.1°, 31.4°, 38.6°, 45.2°, 50.9°, and 56.0°, corresponding to the (001), (110), (111), (002), (220), and (112) reflections respectively. The pyrochlore phase peak (222) at 2θ = 29.4° was clear for samples annealed at 600 °C and 700 °C and was identified as Pb_2_Nb_2_O_7_ by the comparison to JCPDS No. 40-828, which showed the other characteristic diffraction peak at 2θ = 34.1°. Compared to the typical PMN-PT solid solution, the single perovskite structure was obtained at 650 °C, which showed two strong peaks at 2θ = 22.2° and 45.3°, indicating a mainly preferred crystal growth along the (001)_c_ direction. Figure 1c,d present the phase structure of 2.5% Pr-PMN-PT thin films deposited on Pt/TiO_2_/SiO_2_/Si substrates annealed at 650 °C for 1 min, 3 min, and 5 min respectively. It was found that 2.5% Pr-PMN-PT thin films were mostly in the perovskite phase with a little pyrochlore phase when annealed for 1 min, which was further crystallized to a single perovskite phase structure after one more minute annealing. However, a peak of the pyrochlore phase was found again in the sample annealed for 5 min, which can be ascribed to the greater lead loss caused by the longer time annealing [19].

The degree of (001) texture in 2.5% Pr-PMN-PT thin film was evaluated by preferred orientation parameter *α*_hkl_, which can be calculated by the following formula [32]:

(2) $$\alpha_{\mathrm{hkl}}=\frac{I_{\mathrm{hkl}}}{\sum I_{\mathrm{hkl}}},$$

where *I*_hkl_ is the relative intensity of the corresponding diffraction peaks (hkl). As shown in Figure 1a,c, *α*_001_ values are 86.6% and 73.6% in Pr-PMN-PT thin films annealed at 650 °C for 2 and 3 min respectively, presenting the highly (001)-oriented textured structure. The preferred orientation was related to many factors, such as substrate materials [33], growth method, doping elements [31], annealing temperatures [34], and so forth. For Pr-PMN-PT thin films, the nucleation was in random direction at the start of thin film growth on the surface of Pt. With further temperature increases up to 650 °C, the (001) direction became the preferential growth direction.

### 3.2. Surface Morphology and Electric Properties at Room Temperature

**Figure 2:**
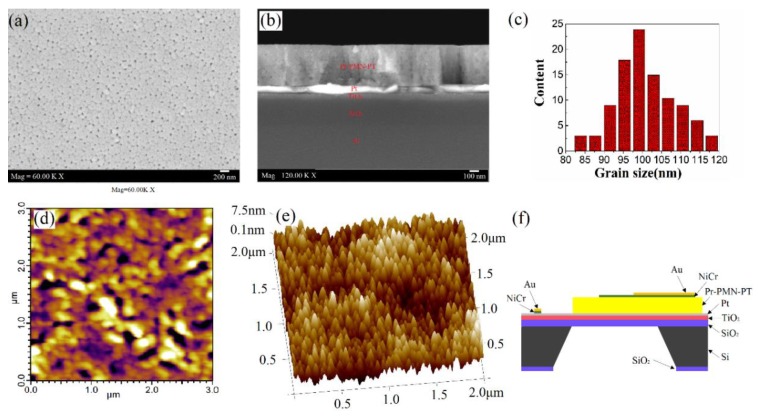
(**a**) FE-SEM images of surface morphologies for 2.5% Pr-PMN-PT thin films annealed at 650 °C for 2 min, (**b**) FE-SEM images of cross-section of 2.5% Pr-PMN-PT thin films, (**c**) grain size distributions of 2.5% Pr-PMN-PT thin films, (**d**) 2D piezoresponse force microscope (PFM) images of surface morphologies for 2.5% Pr-PMN-PT thin films, (**e**) 3D atomic force microscope (AFM) images of surface morphologies for 2.5% Pr-PMN-PT thin films, and (**f**) a schematic illustration of the device for electric properties measurement.

Figure 2a,b shows the FE-SEM images of surface and cross-sectional micrographs of Pr-PMN-PT thin films. From these images, it can be seen that the thin films have a dense structure without any cracks. It can further be seen that the quite uniform Pr-PMN-PT, Pt, and TiO_2_ films are around 450 nm, 80 nm, and 80 nm in thickness respectively, which are in agreement with the values measured by a surface profilometer. Figure 2c presents surface grain size distributions calculated from the image in Figure 2a, showing an average grain size of 100 nm. A typical out-of-plane image with a 3 × 3 μm^2^ scan area at the surface of 2.5% Pr-PMN-PT thin films is shown in Figure 2d. The grain size is about 100 nm, in accordance with the FE-SEM observation. However, nano-domains could not be found in the picture, which can be analyzed by macroscale level ferroelectric properties, similar to the results in Ref. [35,36]. The 3D AFM image of 2.5% Pr-PMN-PT thin films is shown in Figure 2e. The root mean square roughness (*R*_rms_) is about 2.59 nm with a scan area of 2 × 2 μm^2^, indicating low interference of electrode contact in electronic measurement. A schematic of the device for further electric investigation is shown in Figure 2f. Two silver wires for electric measurement were bonded to the surface of the Au electrodes.

The dielectric, pyroelectric, and ferroelectric properties in 2.5% Pr-PMN-PT thin films at room temperature are summarized in Table 1. The values of *ε*_r_, *p*_y_, and *p*_r_ in 2.5% Pr-PMN-PT thin films were on the order of 2400, 167 μC/m^2^K, and 17.3 μC/cm^2^, showing 110%, 123%, and 50% higher factors respectively than those in pure PMN-PT thin films. Compared with PMN-0.30PT grown by a chemical solution deposition (CSD) method [19], the *ε*_r_ in PMN-0.30PT thin films on Si substrate was lower than that of the sample synthesized on perovskite structure single crystal substrates. A similar tendency of pyroelectric property was found in PMN-0.30PT thin film grown on various substrates. The *p*_r_ value in 2.5% Pr-PMN-PT thin films was still higher than that of PMN-0.32PT thin films deposited on Ba_0.5_Sr_0.5_RuO_3_/NdScO_3_ and La_0.5_Sr_0.5_CoO_3_/LaAlO_3_ crystals using pulsed laser deposition. In contrast, the *p*_y_ value showed an opposite trend, which is attributed to the lattice misfit. Compared with PVDF and normal ferroelectric material, the relaxor-PT thin films showed higher dielectric and pyroelectric properties. In contrast, the response time of the PMN-0.30PT pyroelectric sensor was longer than that of PVDF [37] and LiTaO_3_ [38].

**Figure 3:**
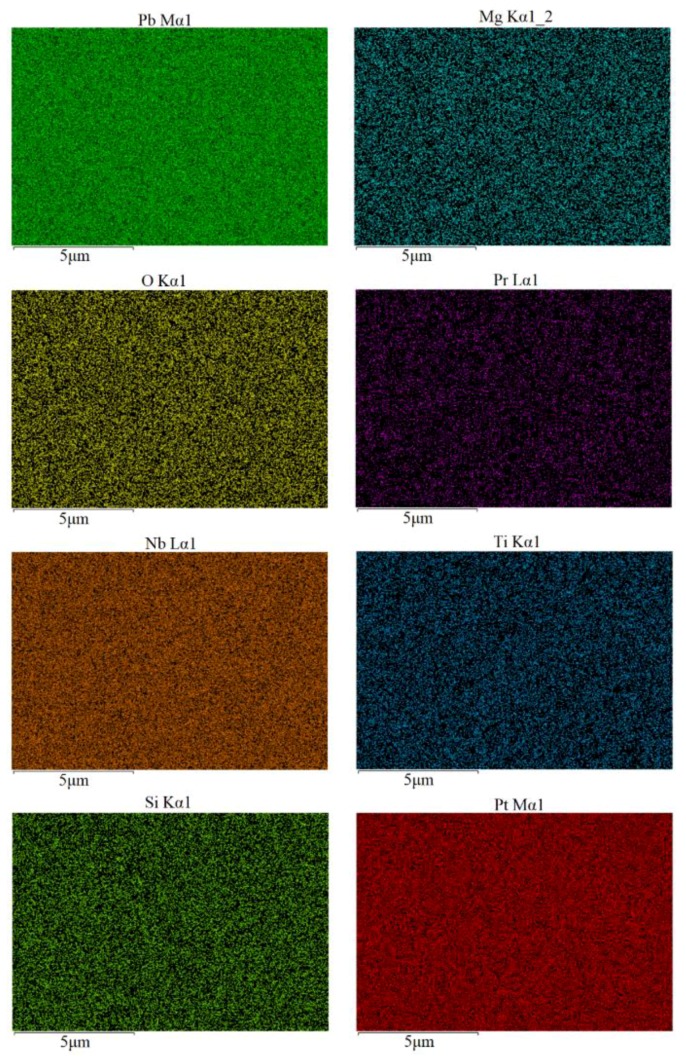
Element mapping of 2.5% Pr-PMN-PT thin films annealed at 650 °C for 2 min.

In order to evaluate the Pr^3+^ doping effect in PMN-PT thin films, the elements distribution was measured, as shown in Figure 3, corresponding to the surface micrograph presented in Figure 2a. The Pb, Mg, O, Pr, Nb, Ti, Si, and Pt maps were achieved by performing standardless quantification of the energy dispersive X-ray spectrometry (EDS) signals in the Lα1 and Kα1 peaks. From these pictures, it can be seen that the Pb, Mg, Nb, Ti, Si, and Pt elements were homogeneously distributed in the local area. It is interesting to note that the Pr element is mostly distributed along the grain boundaries of thin films, compared to Figure 2a.

### 3.3. Ferroelectric Properties

**Figure 4:**
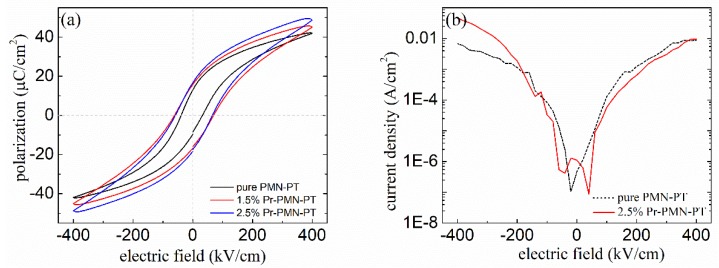
(**a**) P-E hysteresis loops for pure PMN-PT and Pr-PMN-PT thin films annealed at 650 °C for 2 min, (**b**) leakage current density dependent on external electric field for pure PMN-PT and 2.5% Pr-PMN-PT thin films.

Figure 4a presents the ferroelectric properties of Pr-PMN-PT thin films with different Pr^3+^ ratios of 0, 1.5%, and 2.5%. The addition of Pr^3+^ in PMN-PT thin films increased the value of remanent polarization (*P*_r_) and coercive field (*E*_c_) from 11.5 μC/cm^2^ and 35 kV/cm to 17.3 μC/cm^2^ and 63.5 kV/cm respectively. The leakage current densities of pure and 2.5% Pr-PMN-PT thin films were approximately 1.24 × 10^−4^ A/cm^2^ and 5.8 × 10^−5^ A/cm^2^ respectively at 100 kV/cm, as shown in Figure 4b. A donor dopant in PMN-PT samples was poled at the higher electric field due to the higher *E*_c_ in Pr-PMN-PT thin films, indicating that the domain structure was easily reoriented owing to the presence of lead vacancies near the grain boundaries [44,45], corresponding to the Pr^3+^ ion distribution shown in Figure 3. Those effects also led to a higher dielectric constant of Pr^3+^ doped compositions compared to the undoped counterpart, as shown in Table 1.

### 3.4. Temperature Dependent of Dielectric and Pyroelectric Properties

**Figure 5:**
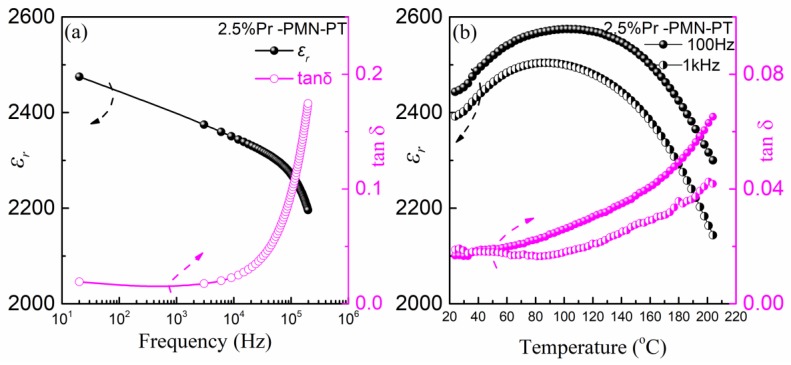
(**a**) Dielectric coefficient (*ε*_r_) and dielectric loss (tanδ) as a function of frequency for 2.5% Pr-PMN-PT thin films annealed at 650 °C for 2 min, (**b**) dielectric coefficient and dielectric loss as a function of temperature for 2.5% Pr-PMN-PT thin films at 100 Hz and 1 kHz.

Figure 5a shows dielectric constant (*ε*_r_) and dielectric loss (tanδ) of Pr-PMN-PT thin films in the frequency range of 20 Hz to 200 kHz at room temperature. We see that the *ε*_r_ value decreased slightly with an increasing frequency, which is induced by the polarization decrease due to charge accumulation at low frequency. Meanwhile, the dielectric loss showed a very quick increase in the range 10^4^–2 × 10^5^ Hz, indicating conduction through the electrode surface and grain boundary capacitance interference in poled samples at high frequency. Figure 5b presents the dielectric properties dependent on temperature for Pr-PMN-PT thin film at 100 Hz and 1 kHz. The *ε*_r_ curve presents a broad peak centered at around 100 °C, which corresponds to the diffuse ferroelectric to paraelectric phase transition [10,17]. The value of dielectric loss values is about 1.8% in the temperature range from 25 to 100 °C, which indicates good electrode bonding for further device integrating.

**Figure 6:**
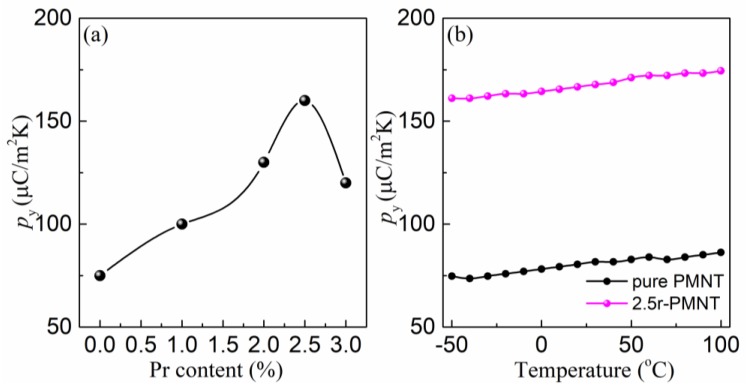
(**a**) Pyroelectric coefficient (*p*_y_) dependent of Pr content in Pr-PMN-PT thin film annealed at 650 °C for 2 min; (**b**) pyroelectric coefficient (*p*_y_) measured in pure PMN-PT and 2.5% Pr-PMN-PT thin films at temperature between −50 °C and 100 °C.

Figure 6a shows the pyroelectric coefficient (*p*_y_) measured at room temperature for the obtained PMN-PT thin films as a function of Pr^3+^ doping content. The *p*_y_ in the thin films with 2.5% dopant presented the maximum value on the order of 167 μC/m^2^K, which is almost a two-fold increase over that of pure PMN-PT thin films, offering a new way for enhancing the pyroelectric performance in relaxor-PT thin films for use in infrared detectors. The temperature dependence of *p*_y_ values is illustrated in Figure 6b. In the temperature range of −50–100 °C, the pyroelectric coefficient showed a slight fluctuation (~10%), indicating good thermal stability for application in infrared detectors integrated into Si semiconductor devices.

## 4. Conclusions

The pure and Pr^3+^ doped PMN-PT ferroelectric thin films with a single perovskite structure were prepared by a sol–gel processing and investigated by X-ray diffraction. The microstructure of Pr-PMN-PT samples was characterized by scanning electron microscopy, atomic force microscopy, and piezoresponse force microscopy. The average grain size was about 100 nm. It was found that Pr^3+^ ions were located at the edge of grain boundaries measured by energy dispersive X-ray spectroscopy. For 2.5% Pr^3+^ doping, the dielectric constant, pyroelectric coefficient, and remenant polarization all reached a maximum. Importantly, the dielectric constant in PMN-PT thin films increased from 1140 to 2400 through 2.5% Pr^3+^ doping. Such enhancement was ascribed to the domain wall motion induced by the Pr^3+^ ion distribution around the grain boundary. Finally, this rare earth dopant presents a useful method for developing high performance relaxor-PT thin films for use in energy transducers and photodetectors.

## Figures and Tables

**Table 1:** Dielectric, pyroelectric, and ferroelectric properties in (001)-oriented PMN-PT based ferroelectric thin films at room temperature.

Thin Films	Substrates	Thickness (nm)	Method	*ε*_r_ _@1kHz_	tanδ (%)	*p*_y_ (μC/m^2^K)	*p*_r_ (μC/cm^2^)	Reference
PMN-0.30PT	Pt/TiO_2_/SiO_2_/Si	450	Sol-gel	1140	3.1	75	11.5	This work
2.5%Pr-PMN-0.30PT	Pt/TiO_2_/SiO_2_/Si	450	Sol-gel	2400	1.5	167	17.3	This work
PMN-0.30PT	Pt/TiO_2_/SiO_2_/Si	350	CSD	1710	0.8	/	/	[20]
PMN-0.32PT	Pt/Si/ITO glass	800	Sol-gel	1110	/	/	0.55	[39]
PMN-0.30PT	SrRuO_3_/SrTiO_3_	200	CSD	~2600	~5	/	~13	[19]
PMN-0.32PT	Ba_0.5_Sr_0.5_RuO_3_/ NdScO_3_	150	PLD	~1200	~7	−550	~8	[4]
PMN-0.32PT	La_0.5_Sr_0.5_CoO_3_/ LaAlO_3_	150	PLD	~2500	~7	160–300	~11	[40]
LiNbO_3_	SiO_2_	325 × 10^3^	/	/	/	40	~50	[41,42]
PVDF	PMMA	28 × 10^3^	/	~5	~2	23	/	[37,43]

*ε*_r_ dielectric constant, tanδ dielectric loss, *p*_y_ pyroelectric coefficient, *p*_r_ remnant polarization, pulsed-laser deposition (PLD), chemical solution deposition (CSD).
